# Successful Transfer of a Model T-DNA Plasmid to *E. coli* Revealed Its Dependence on Recipient RecA and the Preference of VirD2 Relaxase for Eukaryotes Rather Than Bacteria as Recipients

**DOI:** 10.3389/fmicb.2018.00895

**Published:** 2018-05-28

**Authors:** Yuta Ohmine, Kazuya Kiyokawa, Kazuya Yunoki, Shinji Yamamoto, Kazuki Moriguchi, Katsunori Suzuki

**Affiliations:** Department of Biological Science, Graduate School of Science, Hiroshima University, Hiroshima, Japan

**Keywords:** horizontal DNA transfer, conjugation, *recA*, T-DNA transfer, type 4 secretion system, *Agrobacterium*, relaxase, plasmid transfer

## Abstract

In *Agrobacterium*-mediated transformation (AMT) of plants, a single-strand (ss) T-DNA covalently linked with a VirD2 protein moves through a bacterial type IV secretion channel called VirB/D4. This transport system originates from conjugal plasmid transfer systems of bacteria. The relaxase VirD2 and its equivalent protein Mob play essential roles in T-DNA transfer and mobilizable plasmid transfer, respectively. In this study, we attempted to transfer a model T-DNA plasmid, which contained no left border but had a right border sequence as an origin of transfer, and a mobilizable plasmid through the VirB/D4 apparatus to *Escherichia coli, Agrobacterium* and yeast to compare VirD2-driven transfer with Mob-driven one. AMT was successfully achieved by both types of transfer to the three recipient organisms. VirD2-driven AMT of the two bacteria was less efficient than Mob-driven AMT. In contrast, AMT of yeast guided by VirD2 was more efficient than that by Mob. Plasmid DNAs recovered from the VirD2-driven AMT colonies showed the original plasmid structure. These data indicate that VirD2 retains most of its important functions in recipient bacterial cells, but has largely adapted to eukaryotes rather than bacteria. The high AMT efficiency of yeast suggests that VirD2 can also efficiently bring ssDNA to recipient bacterial cells but is inferior to Mob in some process leading to the formation of double-stranded circular DNA in bacteria. This study also revealed that the recipient *recA* gene was significantly involved in VirD2-dependent AMT, but only partially involved in Mob-dependent AMT. The apparent difference in the *recA* gene requirement between the two types of AMT suggests that VirD2 is worse at re-circularization to complete complementary DNA synthesis than Mob in bacteria.

## Introduction

The T-DNA transfer system is derived from bacterial conjugal plasmid DNA transfer systems (Lawley et al., 2004), which exchange genetic material between bacterial species. There are convincing similarities between the T-DNA and bacterial conjugal transfer systems (Lessl and Lanka, 1994). *Agrobacterium* VirD2 relaxase, in collaboration with VirD1, makes a nick at the right border (*RB*) and left border (*LB*) sequences and covalently attaches to the 5′ end of the resulting single-stranded (ss) T-DNA (Ward and Barnes, 1988; De Vos and Zambryski, 1989; Vogel and Das, 1992; Scheiffele et al., 1995). Essentially the same reaction takes place in bacterial conjugation. TraI encoded in the F plasmid binds to and makes a nick at the origin of transfer (*oriT*) with assistance by the TraY protein (Ippen-Ihler and Skurray, 1993). MobA encoded in the mobilizable plasmid RSF1010 recognizes *oriT* as its DNA substrate (Scholz et al., 1989). The TraI and MobA relaxases produce a nick at *oriT* and covalently attach to the 5′ end of the resulting ssDNA of the respective plasmids (Pansegrau and Lanka, 1991; Scherzinger et al., 1993; Bohne et al., 1998; Fullner, 1998). The complexes between the relaxase and ssDNA from each plasmid DNA are transported through a T4SS into recipient cells (Frey and Bagdasarian, 1989; Zambryski, 1992; Firth et al., 1996; Alvarez-Martinez and Christie, 2009; Wong et al., 2012).

The large operon *virB* in Ti plasmids (Suzuki et al., 2009) harbors 11 genes for the formation of a T4SS, called the VirB/D4 channel (Alvarez-Martinez and Christie, 2009). A similar set of genes is dedicated to the construction of a T4SS for the transfer of F and RP4/RK2 plasmids (Lawley et al., 2003; Christie et al., 2014). Transfer via the T4SS requires another factor called a coupling protein, e.g., VirD4 for the T-DNA of Ti plasmids and TraD for F. Specifically, the coupling proteins recognize nucleo-protein substrates and then pass appropriate substrates to the T4SS membrane-spanning channel, and therefore are also called the gatekeepers of the channel (Lawley et al., 2003; Christie et al., 2014).

Conjugal plasmid transfer among Gram-negative bacteria is generally recognized as the integration of four steps, namely the formation and inter-cellular transfer of ssDNA, re-circularization of the transferred DNA and completion of the complementary lagging strand DNA synthesis in recipient bacterial cells (Bhattacharjee and Meyer, 1991). The ssDNAs emerging in the recipient bacterial cytoplasm would bind to SSBs and probably to RecA before the completion of the re-circularization and complementary DNA synthesis. Conjugal transfer is quite similar to T-DNA transfer but has several differences, not only of the relaxases, but also their processes in recipient cells. During T-DNA transfer, VirD2 at the 5′-end of the ssT-DNA should remain intact in the eukaryotic recipient cytoplasm (Gelvin, 2012), while rapid re-circularization in recipient bacterial cells would be required for a high yield of plasmid transconjugants. The ssDNA binding protein VirE2 is essential for plant tumorigenesis by agrobacteria and AMT (Zupan et al., 1996), whereas the significance of plasmid-encoded SSBs remains obscure in conjugal plasmid transfer (Lanka and Pansegrau, 1999).

T-DNA transfer by *Agrobacterium tumefaciens* can genetically transform a broad range of eukaryotic organisms including fungi and mammalian cells under laboratory conditions (Lacroix et al., 2006). This wide transfer range suggests that the factors provided by recipient cells are so conserved that they can associate well with those from *Agrobacterium*. Such exotic combinations of donor and recipient organisms or T4SS and substrate DNAs could give insights into the mechanisms involved. Mobilizable plasmids are delivered through conjugation, though they possess no gene for any membrane-spanning channel (Smillie et al., 2010). Such plasmids employ a carrier T4SS supplied by a conjugative plasmid, e.g., RP4/RK2. Several mobilizable plasmids can also be transferred by the *Agrobacterium* VirB/D4 T4SS, e.g., RSF1010 and pTF-FC2 to plant cells (Bravo-Angel et al., 1999; Dube et al., 2004) and RSF1010 to other *Agrobacterium* cells (Beijersbergen et al., 1992).

At present, little is known about T-DNA transfer to bacteria. Only one paper, by Kelly and Kado (2002), has reported T-DNA transfer to a Gram-positive bacterium, *Streptomyces lividans*. Extensive investigation of T-DNA transfer to bacteria might reveal the differences between the processes of T-DNA transfer and conjugative transfer and how T-DNA transfer evolved to adapt to eukaryotic recipients.

In this study, we constructed a model T-DNA plasmid that contained an *RB*, and attempted VirD2-mediated transfer of this plasmid to bacteria and compared the results with transfer to yeast and with Mob-mediated transfer of a mobilizable plasmid. In the VirD2-driven transport experiments, the recipient *E. coli* exhibited much lower efficiency than yeast. Inversely, in Mob-driven transport, the recipient *E. coli* showed higher efficiency than yeast. These results indicate that the T-DNA transfer system retains the features of conjugal transfer, but has a functional inclination toward eukaryotes.

## Materials and Methods

### Bacterial Strains and Culture Conditions

The bacterial and yeast strains used in this study are listed in **Table 1**. *E. coli* strain BW25113 and a set of knockout mutant derivatives of BW25113 (Baba et al., 2006) were supplied by the National BioResource Project (National Institute of Genetics, Japan). The *recAΔ* mutant in the set was endowed with streptomycin resistance by spontaneous mutation and a kanamycin resistance gene cassette was removed by site-specific recombination using FLP recombinase according to Baba et al. (2006). Yeast cells were cultured in liquid YPD medium at 28°C, while *E. coli* and *Agrobacterium* strains were grown in liquid LB medium at 37°C and 28°C, respectively. Co-cultivation for yeast AMT was performed as described in our previous papers (Kiyokawa et al., 2009, 2012; Ohmine et al., 2016), and is briefly explained below. Co-cultivation for AMT of bacteria was carried out essentially following that for the yeast AMT, with some modifications as mentioned in the corresponding subsection.

**Table 1:** Bacterial and yeast strains, and plasmids used in this study.

Strain or plasmid	Relevant genotype and/or characteristics	Reference or source
***Agrobacterium tumefaciens* strains**		
C58m	Rif^r^ and Nal^r^ mutant of pathogenic strain C58 carrying pTiC58	Our collection
EHA105	C58 containing pTiEHA105 (disarmed pTiBo542)	Hood et al., 1993
EHA105Bo*virD2Δ*	EHA105 with deletion of *virD2* in pTiEHA105	This study
EHA105Bo*vir*E*2Δ*	EHA105 with deletion of *virE2* in pTiEHA105	This study
***Escherichia coli* strains**		
LE392	F^-^ *glnV44 supF58 (lacY1 or lacZYΔ) galK2 galT22 metB1 trpR55 hsdR514(rK-mK+)*	Our collection
LE392Sm	Sm^r^ mutant of LE392	This study
BW25113	F^-^ *rrnB3 lacZ4787Δ hsdR514 (araBAD)567Δ (rhaBAD)568Δ rph-1*	NIG
BW25113sm	Sm^r^ mutant of BW25113	This study
HB101	*F^-^ mcrB mrr hsdS20(rB^-^ mB^-^) recA13 leuB6 ara-14 proA2 lacY1 galK2 xyl-5 mtl-1 glnV44aaa* Sm^r^	Boyer and Roulland-Dussoix, 1969
DHI0B	F^-^ *endA1 deoR^+^ recA1 galE15 galK16 nupG rpsL (lac)X74Δ (φ80lacZΔM15 araD139 (ara, leu) 7697Δ mcrA (mrr-hsdRMS-mcrBC) Δ aaa* Sm^r^	Grant et al., 1990
S17-1bbbpir	F^-^ *thi pro hsdR [RP4-2 Tc::Mu Km::Tn7 (Tp Sm)] bbbpir* Tp^r^ Sm^r^	Simon et al., 1983
BW25113*recAΔ*	*recAΔ* mutant in *E. coli* single-gene knockout mutant collection (Keio collection) Km^r^	Baba et al., 2006
BW25113*recAΔ* sm	Sm^r^ mutant of *BW25113recAΔ*	This study
***Saccharomyces cerevisiae* Strain**		
BY4742	*MATα his3Δ1 leu2Δ0 lys2Δ0 ura3Δ0*	Brachmann et al., 1998
**Plasmids**		
pAY205	*ARS1, TRP1, URA3, oriV^incQ^, oriT^incQ^, mob^incQ^* and Km^r^	Nishikawa et al., 1992
pSRKKm	*mob^pBBR1^, oriT^pBBR1^, rep^pBBR1^* and Km^r^	Khan et al., 2008
pBIN19	Binary vector with *nptII* driven by Pnos; Km^r^	Bevan, 1984
pRS316	*URA3, ARSH4/CEN6* and Amp^r^ (Car^r^)	Sikorski and Hieter, 1989
pSRK-R316	*RB, oriT^pBBR1^, rep*^pBBR1^, *URA3, ARSH4/CEN6*, Km^r^ and Amp^r^ (Car^r^)	This study
pSRK-R316ΔRB	*oriT^pBBR1^, rep^pBBR1^, URA3, ARSH4/CEN6*, Km^r^ and Amp^r^ (Car^r^)	This study
pK18mobsacB	*pMB1 ori, oriT^incP^, sacB*, Km^r^	Schäfer et al., 1994
pK18msΔBovirE2	pK18mobsacB containing upstream and downstream of *virE2* derived from pTiEHA105; Km^r^	This study
pK18msΔBovirD2	pK18mobsacB containing upstream and downstream of *virD2* derived from pTiEHA105; Km^r^	This study

### Plasmid Construction

The plasmids and primers used in this study are listed in **Tables 1, 2**, respectively. The binary plasmid pSRK-R316 was constructed as follows. pSRKKm (Khan et al., 2008) was digested with *Csp*45I and the resulting 4.1-kbp DNA fragment lacking the *lacI^q^* and *lacZ* genes was self-ligated to produce pSRK-Csp. A 3.0-kbp DNA fragment, lacking the *mob* gene, was amplified using the primers pSRKKm-rep-fw2 and pSRKKm-Km-rv with pSRK-Csp as a template. The resulting 3.0-kbp PCR product was digested with *Spe*I and then self-ligated to form the plasmid pSRK-KR. A 2.9-kbp fragment was amplified by PCR using the primers pSRK-C-fwS and pSRK-KR-rv from the plasmid pSRK-KR. In addition, a 0.2-kbp fragment, containing the *RB* and overdrive sequences, was amplified using the primers pBIN19-RB-fw and pBIN19-RB-rv from the binary vector pBIN19 (Bevan, 1984). The resulting 2.9-kbp and 0.2-kbp PCR products were digested with *Bam*HI and *Sac*II, and then ligated to each other to construct the 3.1-kbp plasmid pSRK-RB. A 3.7-kbp DNA was amplified by PCR using pRS316-fwB and pRS316-rvB from the yeast–*E. coli* shuttle vector pRS316 (Sikorski and Hieter, 1989). The PCR product and the plasmid pSRK-RB were digested with *Bam*HI and then ligated together to produce pSRK-R316.

**Table 2:** Oligonucleotide primers used in this study.

Primer	Resultant construct	Sequence (5′-3′)
pSRKKm-rep-fw2	pSRK-R316 and pSRK-R316ΔRB	GACTAGTTGGTGTCCAACCGGCTCGACG
pSRKKm-Km-rv	pSRK-R316 and pSRK-R316ΔRB	GACTAGTCTCGAGGCAGTGGGCTTACATGGCGATAG
pSRK-C-fwS	pSRK-R316 and pSRK-R316ΔRB	TCCCCGCGGTCACACTGCTTCCGGTAGTCA
pSRK-KR-rv	pSRK-R316 and pSRK-R316ΔRB	CGGGATCCCGGCTTCCATTCAGGTCGAG
pBIN19-RB-fw	pSRK-R316 and pSRK-R316ΔRB	CGGGATCCTGACAGGATATATTGGCGGGTAAACC
pBIN19-RB-rv	pSRK-R316 and pSRK-R316ΔRB	TCCCCGCGGCCAATCTTGCTCGTCTC
pRS316-fwB	pSRK-R316 and pSRK-R316ΔRB	CGGGATCCGTTAAGGGATTTTGGTCATGAGA
pRS316-rvB	pSRK-R316 and pSRK-R316ΔRB	CGGGATCCACATGTTCTTTCCTGCGTTAT
pSRK-C-fwEH	pSRK-R316ΔRB	GGAATTCAAGCTTGGTCACACTGCTTCCGGTAGTCA
pRS316-fwKH	pSRK-R316ΔRB	GGGGTACCAAGCTTGTTAAGGGATTTTGGTCATGAGA
T-circle-fw	for sequencing	TCATGTAACTCGCCTTGATCGTT
T-circle-rv	for sequencing	GATGCCTGCTTGCCGAATATC
virD2Bo542-del-Fw1	pK18msΔBovirD2	ATGACATGATTACGAATTCTCACGTTGCTGGTCTTTCTC
virD2Bo542-del-Rv1	pK18msΔBovirD2	GTGAACTGACCATTTGCCATCCAATTTTCTCCCGTCAGGTG
virD2Bo542-del-Fw2	pK18msΔBovirD2	ATGGCAAATGGTCAGTTCAC
virD2Bo542-del-Rv2	pK18msΔBovirD2	TGCCAAGCTTGCATGCCTGCAGACAGAGGTGTACGATGTCAG
virE2Bo542-del-Fw1	pK18msΔBovirE2	ATGACATGATTACGAATTCAAGGCGACTGTTGCTTAACG
virE2Bo542-del-Rv1	pK18msΔBovirE2	CGTCTCACTCCTTCTGACCAG
virE2Bo542-del-Fw2	pK18msΔBovirE2	GTCAGAAGGAGTGAGACGATGGTGAACACTACAAAGAAAAG
virE2Bo542-del-Rv2	pK18msΔBovirE2	TGCCAAGCTTGCATGCCTGCAGGATTGTCCGAGGATGAAGAC

The plasmid pSRK-R316ΔRB was prepared as follows. A 5.5-kbp DNA fragment, lacking the *RB* sequence, was amplified using the primers pSRK-C-fwEH and pRS316-fwKH from pSRK-R316. The resulting 5.5-kbp PCR product was digested with *Hind*III and then self-ligated, resulting in the *RB*-free plasmid pSRK-R316ΔRB.

Plasmids for targeted gene deletion were created by seamless fusion (Motohashi, 2015; Okegawa and Motohashi, 2015) between the *Eco*RI/*Pst*I-digested pK18mobsacB and two DNAs located upstream and downstream of the ORF, which were amplified by PCR using the primer sets virD2Bo542-del-Fw1 and virD2Bo542-del-Rv1, and virD2Bo542-del-Fw2 and virD2Bo542-del-Rv2 to form an 8.8-kbp plasmid, pK18msΔBovirD2; and virE2Bo542-del-Fw1 and virE2Bo542-del-Rv1, and virE2Bo542-del-Fw2 and virE2Bo542-del-Rv2 to form an 8.8-kbp plasmid, pK18msΔBovirE2.

Targeted gene replacement *in vivo* by HR was carried out as described previously (Kiyokawa et al., 2012).

#### PCR

Amplification by PCR was carried out using KOD Plus NEO DNA polymerase (TOYOBO, Osaka) for plasmid DNA construction and DNA preparation for transformation.

#### *Agrobacterium*-Mediated Transformation of Yeast and Bacterial Strains

AMT of the yeast strain was performed as described in our previous papers (Kiyokawa et al., 2009, 2012; Ohmine et al., 2016). In short, *Agrobacterium* donor cells were pre-treated with liquid AB induction medium containing 100 μM AS at 28°C for 24 h, and then co-cultivated with recipient yeast cells on solid AB mating medium containing 100 μM AS for 24 h at 22°C. Yeast AMT transformants were selected using a solid SD selective medium (without uracil) containing 200 μg/ml cefotaxime.

AMT of Gram-negative bacteria was performed as follows. Aliquots of 50 μl of donor cell suspension (1.2 × 10^10^ cells/ml) and recipient cell suspension (2.4 × 10^9^ cells/ml) were mixed and then spotted on solid AB induction medium containing AS. After co-cultivation for 24 h at 22°C, the cell mixture was resuspended in AB medium (pH 7.0) and then spread on solid LB medium supplemented with 50 μg/ml kanamycin and 400 μg/ml streptomycin to select for AMT transformant colonies. The AMT efficiency was calculated by dividing the AMT transformant colony number by the output recipient cell number.

### Bacterial Transformation

Circular and linear DNAs, namely intact pSRK-R316 and its PCR product amplified using the primers pBIN19-RB-fw and pRS316-fwKH (the PCR product was a linear double-stranded DNA containing approximately the whole plasmid sequence), were transformed into *E. coli* strains by electroporation as described previously (Sawahel et al., 1993; Yamamoto et al., 2007).

### Sequence Analysis of Transferred Plasmids

The transferred pSRK-R316 plasmids were extracted from each transformant colony. The region around the *RB* sequence was amplified by PCR using the primers T-circle-fw and T-circle-rv. The PCR products were applied to sequencing reactions and analyzed with a Genetic Analyzer 3130XL (Applied Biosystems).

### Statistical Analysis

All experiments in this study were independently repeated at least three times. Each datum shown in figures and tables represents a mean with a standard deviation. Statistical analyses were carried out using the R program version 3.3.3 and its expansion packages^1^. Individual methods for statistical comparisons are described in each table and figure. Data of no AMT colony were excluded from the statistical analyses.

## Results

**FIGURE 1:**
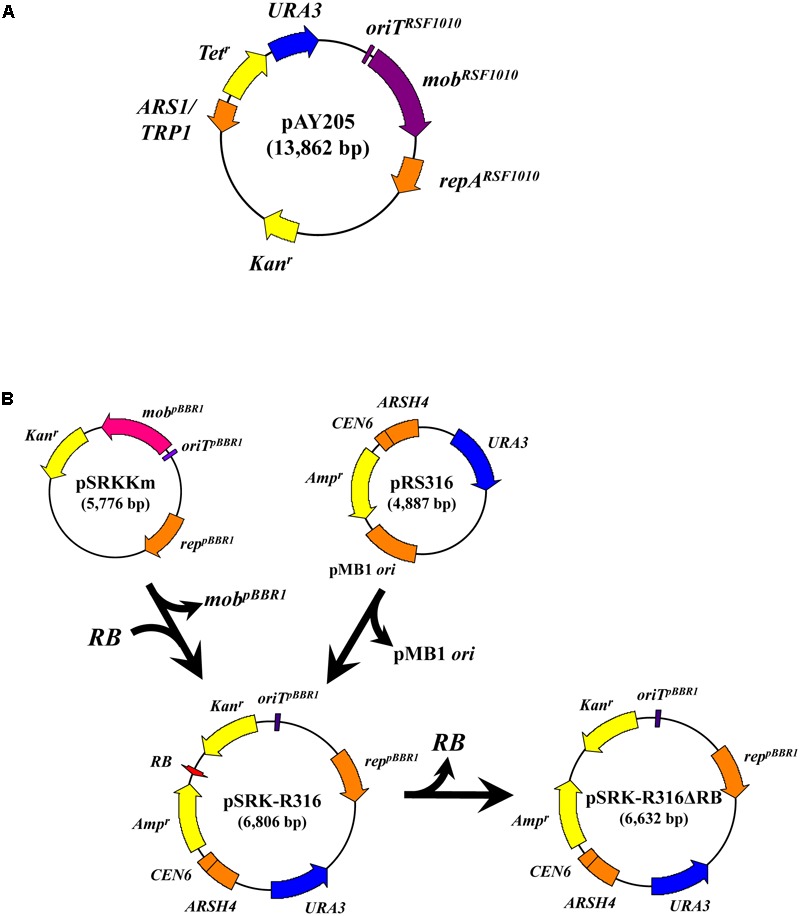
Construction of the binary plasmid pSRK-R316 and its *RB*-less derivative. **(A)** Schematic map of pAY205. **(B)** Most of pSRKKm excluding the *mob^pBBR1^* gene was PCR amplified and ligated with an *RB* fragment derived from pBIN19 as described in the Section “Materials and Methods.” The resulting plasmid was ligated with the yeast autonomous-type vector pRS316 to produce pSRK-R316. pSRK-R316ΔRB is pSRK-R316 but lacks the *RB*.

We performed AMT using *Agrobacterium* strain C58m and two *E. coli* strains, LE392sm and BW25113sm, as recipients. In this experiment, the donor *Agrobacterium* strain EHA105 was loaded with two types of plasmids. As shown in **Figure 1A**, the IncQ plasmid pAY205, which is a derivative of the broad-host-range plasmid RSF1010, encodes the *mob* gene and *oriT* of RSF1010 (*mob^RSF1010^* gene and *oriT ^RSF1010^*) (Nishikawa et al., 1992). The broad-host-range plasmid pSRKKm (**Figure 1B**) is a pBBR1-based plasmid and encodes the *mob* gene and *oriT* of pBBR1 (*mob^pBBR1^* gene and *oriT ^pBBR1^*) (Khan et al., 2008). The new plasmid pSRK-R316 (**Figure 1B**) is a pSRKKm-derivative plasmid. pSRK-R316 lacks the *mob* gene but contains an *RB* and overdrive sequence set derived from the binary plasmid pBIN19 (Bevan, 1984). The *RB* and overdrive sequence set is abbreviated to *RB* in this paper. Therefore, pSRK-R316 was expected to be recognized by VirD2 and transported through the VirB/D2 channel.

### Successful Transfer of the Model T-DNA Plasmid to *E. coli* and *Agrobacterium*

**FIGURE 2:**
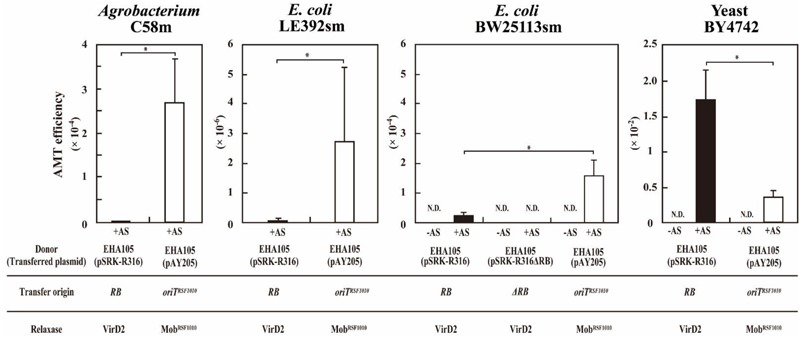
*Agrobacterium*-mediated transformation of a yeast and two bacteria. Recipient cells were co-cultivated with *Agrobacterium* strain EHA105 (pSRK-R316) (filled bar) and with EHA105 (pAY205) (open bar). The AMT efficiency was defined as the number of AMT transformants divided by the number of output recipient cells. Recipient strains included *A. tumefaciens* strain C58m, *E. coli* strains LE392sm and BW25113sm, and yeast strain BY4742. Single asterisks indicate significant difference (*P* < 0.05) by Student’s *t*-test.

In the test of DNA transfer to bacteria, mobilization of pAY205 to *Agrobacterium* strain C58m occurred at high efficiency (2.7 × 10^-4^) (**Table 3A**). However, when incubated with the donor *Agrobacterium* containing pSRK-R316, C58m produced only a few offspring colonies at an efficiency of 2.3 × 10^-7^ (**Figure 2** and **Table 3A**). Similar results with lower efficiencies were obtained when *E. coli* strain LE392sm was employed as a recipient (**Figure 2** and **Table 3A**), whereas the experiment using *E. coli* strain BW25113sm as a recipient produced many more transformant colonies than that using LE392sm. The AMT efficiency of BW25113sm for pSRK-R316 reached an order of 10^-5^ (2.4 × 10^-5^) (**Figure 2** and **Table 3A**).

**Table 3:** AMT of Gram-negative bacteria and a yeast.

(A)
Transferred plasmid (Relevant characteristics)	Recipient	AMT efficiency^a^		%pSRK-R136 transfer With AS
		Without AS	With AS	
	***A. tumefaciens***			
pSRK-R316 (*RB, oriT^pBBR1^*)	C58m	NT^b^	(2.3 ± 0.2) × 10^-7^	100
pAY205 (*oriT^RSF1010^, mob^RSF1010^*)	C58m	NT^b^	(2.7 ± 0.9) × 10^-4 ∗c^	117391

	***E. coli***			
pSRK-R316 (*RB, oriT^pBBR1^*)	LE392sm	NT^b^	(5.9 ± 4.8) × 10^-8^	100
pAY205 (*oriT^RSF1010^, mob^RSF1010^*)	LE392sm	NT^b^	(2.7 ± 2.5) × 10^-6 ∗c^	4576
pSRK-R316 (*RB, oriT^pBBR1^*)	BW25113sm	<(9.3 ± 1.3) × 10^-8^	(2.4 ± 1.1) × 10^-5^	100
pSRK-R316ΔRB (*oriT^pBBR1^*)	BW25113sm	<(6.7 ± 0.1) × 10^-8^	<(5.8 ± 1.0) × 10^-7^	0
pAY205 (*oriT^RSF1010^, mob^RSF1010^*)	BW25113sm	<(1.9 ± 0.6) × 10^-7^	(1.6 ± 0.5) × 10^-4 ∗c^	667

	**Yeast**			
pSRK-R316 (*RB, oriT^pBBR1^*)	BY4742	<(1.8 ± 0.6 × 10^-5^)	(1.7 ± 0.6) × 10^-2^	100
pAY205 (*oriT^RSF1010^, mob^RSF1010^*)	BY4742	<(1.7 ± 0.1 × 10^-5^)	(3.6 ± 1.3) × 10^-3 ∗c^	21

**(B)**
**Donor *Agrobacterium* (Transferred plasmid)**	**Relevant genotype and characteristics in donor**	**Recipient**	**AMT efficiency^a^ With AS**	**%WT**

		***E. coli***		
WT (pSRK-R316)	*virE2^+^, virD2^+^* (*RB, oriT^pBBR1^*)	BW25113sm	(2.7 ± 1.2) × 10^-5^	100
*virE2Δ* (pSRK-R316)	*virE2Δ, virD2^+^* (*RB, oriT^pBBR1^*)	BW25113sm	(1.8 ± 0.6) × 10^-5^	66
*virD2Δ* (pSRK-R316)	*virE2^+^, virD2Δ* (*RB, oriT^pBBR1^*)	BW25113sm	<(3.5 ± 0.9) × 10^-9^	0
WT (pAY205)	*virE2^+^, virD2^+^* (*oriT^RSF1010^, mob^RSF1010^*)	BW25113sm	(1.5 ± 0.7) × 10^-4^	100
*virE2Δ* (pAY205)	*virE2Δ, virD2^+^* (*oriT^RSF1010^, mob^RSF1010^*)	BW25113sm	(1.2 ± 0.5) × 10^-4^	80
*virD2Δ* (pAY205)	*virE2^+^, virD2Δ* (*oriT^RSF1010^, mob ^RSF1010^*)	BW25113sm	(1.2 ± 0.8) × 10^-4^	80

		**Yeast**		
WT (pSRK-R316)	*virE2^+^, virD2^+^* (*RB, oriT^pBBR1^*)	BY4742	(3.9 ± 0.5) × 10^-3^	100
*virE2Δ* (pSRK-R316)	*virE2Δ, virD2^+^* (*RB, oriT^pBBR1^*)	BY4742	(1.4 ± 0.6) × 10^-3 ∗∗d^	36
*virD2Δ* (pSRK-R316)	*virE2^+^, virD2Δ* (*RB, oriT^pBBR1^*)	BY4742	<(3.0 ± 0.4) × 10^-7^	0
WT (pAY205)	*virE2^+^, virD2^+^* (*oriT^RSF1010^, mob^RSF1010^*)	BY4742	(1.7 ± 0.5) × 10^-4^	100
*virE2Δ* (pAY205)	*virE2Δ, virD2^+^* (*oriT^RSF1010^, mob^RSF1010^*)	BY4742	(5.8 ± 2) × 10^-5 ∗∗d^	34
*virD2Δ* (pAY205)	*virE2^+^, virD2Δ* (*oriT^RSF1010^, mob^RSF1010^*)	BY4742	(1.2 ± 0.1) × 10^-4^	71

*The donor *Agrobacterium* strain EHA105 was loaded with the plasmids as transfer substrates. Mutant strains EHA105Bo*virE2Δ* and EHA105Bo*virD2Δ* were also used instead of EHA105. ^*a*^Data are expressed as output Km^*r*^ colony number (for bacteria) or Ura^+^ colony number (for yeast) per output recipient colony number. ^*b*^Not tested. ^*c*^Single asterisks indicate significant difference (*P* < 0.05) by Student’s *t* -test against pSRK-R316 as transfer substrates in each recipient strain. ^*d*^Double asterisks indicate significant difference (*P* < 0.01) by Dunnett’s test against Wild-type donor strain harboring either pSRK-R316 or pAY205.*

### Reverse Fitness of AMT to Recipient Organisms Depending on VirD2 and Mob

Next, we tested the AMT ability of the plasmids pSRK-R316 and pAY205 using yeast as a eukaryotic recipient. As shown in **Figure 2**, high AMT efficiencies were achieved not only with pAY205 but also pSRK-R316. pSRK-R316 exhibited a fivefold higher efficiency (1.7 × 10^-2^) than pAY205 did (3.6 × 10^-3^) (**Figure 2** and **Table 3A**).

The AMT efficiency of the Mob-driven transfer (pAY205) was 6-fold and 46-fold higher than that of the VirD2-driven transfer (pSRK-R316) when the recipient cells were *E. coli* BW25113sm and LE392sm, respectively (**Table 3A**). These AMT data of the bacterial recipients were contrary to the AMT data of the yeast strain (**Figure 2** and **Table 3A**). The AMT efficiency of Mob-driven transfer (pAY205) was fivefold less than that of VirD2-driven transfer (pSRK-R316) when the recipient cells were yeast. When the VirD2-mediated AMT efficiency was normalized by dividing by the Mob-mediated AMT efficiency in each recipient species, VirD2-mediated AMT of yeast was superior by 33-fold to that of *E. coli* BW25113sm. AMT of *Agrobacterium* strain C58sm was inferior by more than 100-fold to that of BW25113sm.

In this study, AMT efficiency was calculated by dividing the AMT transformant colony number by the output recipient cell number. To confirm the reliability of the formulas used to evaluate the VirD2 and Mob relaxases, we repeated the above experiments but measured input cell numbers and output donor cell numbers in addition to the output recipient cell number. As shown in **Table 4**, calculations using other denominator factors including the square root of (donor number × recipient number) (Simonsen et al., 1990) consistently demonstrated the preference of VirD2-driven transport for yeast, similar to calculations using the standard formulas.

**Table 4:** AMT efficiency calculated by different formulas.

		AMT efficiency									
Donor (Transferred plasmid: Relevant characteristics)	Recipient	AMT colony number divided by output recipient colony number		AMT colony number divided by input donor colony number		AMT colony number divided by input recipient colony number		AMT colony number divided by SquareRoot (input donor colony number × input recipient colony number)		AMT colony number divided by SquareRoot (output donor colony number × output recipient colony number)	
			%pSRK-R136 transfer		%pSRK-R136 transfer		%pSRK-R136 transfer		%pSRK-R136 transfer		%pSRK-R136 transfer
	***E. coli***										
EHA105 (pSRK-R316: *RB, oriT^pBBR1^*)	BW25113sm	(1.2 ± 0.5) × 10^-5^	100	(4.3 ± 2.2) × 10^-6^	100	(4.7 ± 3.6) × 10^-5^	100	(1.4 ± 0.9) × 10^-5^	100	(9.1 ± 43) × 10^-6^	100
EHA105 (pAY205: *oriT^RSF1010^, mob^RSF1010^*)	BW25113sm	(6.6 ± 1.0) × 10^-5 ∗∗b^	550	(3.9 ± 0.6) × 10^-5 ∗∗b^	910	(2.8 ± 0.9) × 10^-4 ∗b^	596	(1.0 ± 0.2) × 10^-4 ∗b^	714	(6.2 ± 0.3) × 10^-5 ∗∗∗b^	681

	**Yeast**										
EHA105 (pSRK-R316: *RB, oriT^pBBR1^*)	BY4742	(1.9 ± 0.6) × 10^-2^	100	(9.5 ± 1.2) × 10^-5^	100	(2.8 ± 0.6) × 10^-2^	100	(1.6 ± 0.2) × 10^-3^	100	(1.1 ± 0.2) × 10^-3^	100
EHA105 (pAY205: *oriT^RSF1010^, mob^RSF1010^*)	BY4742	(1.5 ± 0.8) × 10^-3 ∗b^	7.9	(3.6 ± 2.2) × 10^-5 ∗b^	38	(8.3 ± 4.7) × 10^-3 ∗∗b^	30	(5.4 ± 3.1) × 10^-4 ∗∗b^	34	(2.3 ± 1.3) × 10^-4 ∗∗b^	21
VirD2’s yeast preference index^a^	70	24	20	21	32

*^*a*^VirD2’s yeast preference index normalized by Mob’s performance (AMT efficiency of Mob-driven transfer to bacterium/AMT efficiency of VirD2-driven transfer to bacterium)/(AMT efficiency of Mob-driven transfer to yeast/AMT efficiency of VirD2-driven transfer to yeast) = Mob’s bacterium preference index normalized by VirD2’s performance (AMT efficiency of VirD2-driven transfer to yeast/AMT efficiency of Mob-driven transfer to yeast)/(AMT efficiency of VirD2-driven transfer to bacterium/AMT efficiency of VirD2-driven transfer to bacterium). ^*b*^Single, double, and triple asterisks indicate significant difference (*P* < 0.05, *P* < 0.01, *P* < 0.001, respectively) by Welch’s *t*-test against pSRK-R316 as transfer substrates in each recipient strain.*

Consequently, we conclude that VirD2 is superior to Mob in AMT of yeast, and *vice versa* Mob is better than VirD2 in AMT of bacteria.

### Involvement of VirD2, *RB* and Virulence Gene Expression in Transfer of the Model T-DNA Plasmid to *E. coli* and Yeast

The AMT of *E. coli* strains depended on Vir proteins because no transformant colonies appeared when the inducer chemical AS for expression of *vir* genes was omitted from the co-cultivation medium (**Table 3A**). Although pSRK-R316 lacked a *mob ^pBBR1^* gene, it still contained an *oriT ^pBBR1^* in addition to an *RB*. The region around the *oriT* site was required for the stable replication of pSRK-R316 in the *Agrobacterium* cells (data not shown); thus, the site could not be eliminated. To confirm whether pSRK-R316 was genuinely transferred in an *RB*-dependent manner, we constructed pSRK-R316ΔRB, which was pSRK-R316 lacking the *RB* but retaining *oriT^pBBR1^*. When pSRK-R316ΔRB was used in the transfer experiment to the *E. coli* BW25113sm strain, no transformant colony appeared (**Table 3A**). Furthermore, a *virD2Δ* mutant and a *virE2Δ* mutant were used in the AMT test to determine whether the T-complex component proteins VirD2 and VirE2 are important for AMT to bacteria as well as AMT to yeast. As expected, the *virD2Δ* mutation in the donor *Agrobacterium* cells resulted in inability to transform *E. coli* using pSRK-R316, while the same mutation had a negligible effect (20% reduction) on AMT with pAY205 (**Table 3B**). These results demonstrated that AMT of *E. coli* by pSRK-R316 requires *RB* on the plasmid and VirD2 protein. In addition, the AMT of *E. coli* with pSRK-R316 and pAY205 occurred in a completely AS-dependent manner (**Table 3A**). These data demonstrated that the two plasmids were mobilized through the VirB/D4 T4SS not only in the transfer to yeast but also to *E. coli*, and that the transfer of pSRK-R316 was driven not by Mob^pBBR1^, but by VirD2, while that of pAY205 was executed by Mob^RSF1010^.

### Limited Effect of *virE2* Null Mutation on the Transfer of Plasmids

As shown above, Mob^RSF1010^-driven transfer was apparently less efficient than VirD2-driven transfer to the yeast recipient (**Figure 2** and **Table 3**). Conversely, the AMT efficiency of Mob^RSF1010^-driven transfer was obviously higher than that of VirD2-driven transfer to bacterial recipients (**Table 3A**). One feasible explanation for the lower efficiency of VirD2-driven AMT than Mob-driven AMT of yeast is suppression of VirE2 protein export to recipients by RSF1010-derived plasmids in the donor *Agrobacterium* cells (Binns et al., 1995; Stahl et al., 1998; Bravo-Angel et al., 1999). Supply of VirE2 is required for efficient AMT of yeast (Bundock et al., 1995; Kiyokawa et al., 2012), and a prerequisite for AMT of plants (Binns et al., 1995). Therefore, the RSF1010-derived plasmid pAY205 might decrease its own MobA-driven AMT efficiency of yeast due to decreased VirE2 transport.

To check the validity of the above presumption, we examined the effect of a null mutation in the *virE2* gene. As shown in **Table 3B**, however, *virE2Δ* mutation in the donor *Agrobacterium* strain barely affected AMT of *E. coli* BW25113 in either of the two types of transfer. Conversely, the same mutation decreased the AMT efficiency of yeast to one-third in both types of transfer.

Even in the absence of VirE2, therefore, replacement of the yeast recipient with a bacterial one increased the AMT efficiency of Mob-driven transfer and decreased the efficiency of VirD2-driven transfer (**Table 3B**). In conclusion, the decreased VirE2 supply due to the presence of the RSF1010-derived plasmid in the donor cells has little effect, if any, on AMT of bacteria and a limited effect on AMT of yeast.

### Whelming Importance of *recA* Gene in Recipient Cells for VirD2-Driven AMT, and Less but Significant Importance for Mob-Driven AMT

**FIGURE 3:**
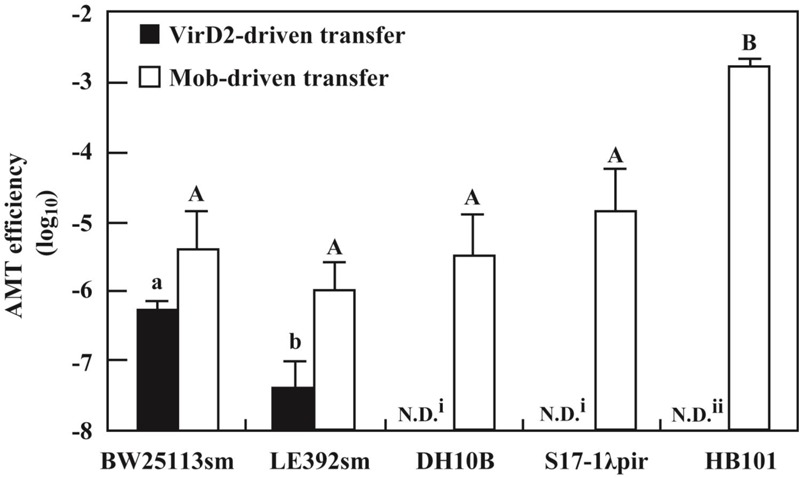
Variation of AMT efficiency among *E. coli* laboratory strains. AMT was applied to five *E. coli* laboratory strains as shown in **Figure 2**. The donor strains used were EHA105 (pSRK-R316) (filled bar) and EHA105 (pAY205) (open bar). N.D. means no transformed offspring were detected (i < 1 × 10^-8^, ii < 4 × 10^-6^). Different letters indicate significant difference (*P* < 0.05) by the Tukey–Kramer test.

As shown above, *E. coli* strains BW25113sm and LE392sm were competent to receive pSRK-R316 from the *Agrobacterium* donor. However, the AMT efficiencies of the two strains differed by more than 10-fold. Therefore, we applied the VirD2-driven transfer system to other *E. coli* strains. As shown in **Figure 3**, the DH10B strain was incompetent for AMT of pSRK-R316. As DH10B is a *recA*-deficient mutant, and BW25113 and LE392 are *recA^+^*, the trial was extended to two more *recA*^-^ mutant strains, S17-1bbb*pir* and HB101. Similar to DH10B, both strains were apparently unsuitable as recipients for VirD2-driven AMT (**Figure 3**).

**FIGURE 4:**
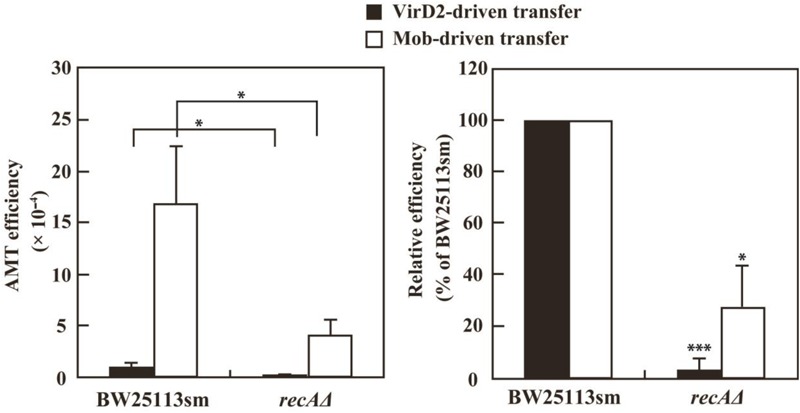
AMT efficiency of a *recA*Δ mutant derivative of the *E. coli* BW25113 strain. BW25113sm (*recA^+^*) and its *recA*Δ mutant strain were subjected to AMT. **(Left)** AMT efficiency. Single asterisks indicate significant difference (*P* < 0.05) by either Welch’s *t*-test (for pSRK-R316 as the transferred substrate) or Student’s *t*-test (for pAY205 as the transferred substrate). **(Right)** Relative efficiency calculated using the AMT efficiency of BW25113sm as a benchmark. Single and triple asterisks indicate that the averages of the relative efficiencies were not 100% (*P* < 0.05, *P* < 0.001, respectively) by one-sample *t*-test.

The defectiveness of the *recA^-^* strains suggested the involvement of the *recA* gene in recipient cells for VirD2-driven AMT in *E. coli*. This idea was confirmed by experiments using a *recAΔ* derivative of the BW25113 strain. As indicated in **Figure 4**, VirD2-driven AMT was 32-fold less efficient in the *recAΔ* strain than in BW25113sm.

In contrast to the large variation among the laboratory *E. coli* strains due to the *recA-*dependence of VirD2-driven transfer, all five *E. coli* strains were apparently competent for Mob-driven AMT (**Figure 3**). All strains except HB101 exhibited efficiencies ranging from 10^-6^ to 10^-5^. HB101 showed a much higher efficiency that reached 10^-3^ (**Figure 3**).

As shown in **Figure 4**, the *recAΔ* mutant of the BW25113 strain showed a threefold lower Mob-driven AMT efficiency than the wild type strain. The ratio was much lower than that (32-fold) of VirD2-driven AMT, but still apparent. This finding suggests some role for the RecA protein even in Mob-driven AMT.

### Evaluation of Two *recA^+^ E. coli* Strains by DNA Transformation

**FIGURE 5:**
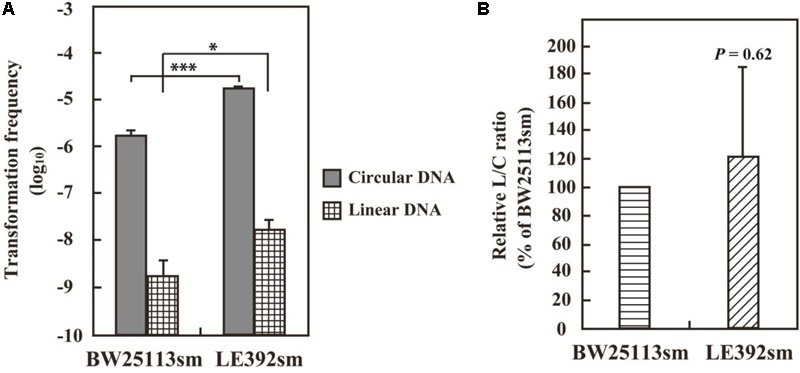
DNA transformation frequencies of *E. coli* strains BW25113sm and LE392sm. *E. coli* cells were electroporated with 50 ng circular pSRK-R316 and linear pSRK-R316 DNA fragments amplified by PCR. Transformation frequency **(A)** is defined as the number of transformants per μg DNA divided by the viable cell number. Single and triple asterisks indicate significant difference (*P* < 0.05 and *P* < 0.001, respectively) by Welch’s *t*-test. **(B)** The L/C transformation ratio was expressed as the ratio of the transformation frequency obtained with the linearized plasmid (L) divided by that using the circular plasmid (C). Statistical analysis was carried out using one-sample *t*-test against 100%.

VirD2-driven AMT was successfully carried out using *E. coli* strain BW25113sm. Apparent, but less efficient, AMT was observed when BW25113sm was replaced with LE392sm. According to their genotypes (**Table 1**), there was no difference in genes that might affect DNA and cellular processes such as DNA repair and modification. Their plasmid DNA transformation ability was measured to see whether the two strains had any difference in their ability to block foreign DNAs. Electroporation was carried out using intact pSRK-R316 and its PCR product as circular and linear DNA substrates, respectively. The latter was a blunt-ended dsDNA containing almost the entire plasmid sequence. As shown in **Figure 5**, the transformation frequency of LE392sm was approximately 10-fold higher than that of BW25113sm for both DNA substrates. When the transformation frequency of the linear DNA substrate (L) was normalized to that of the circular DNA substrate (C), the resulting linear versus circular (L/C) ratio was comparable between the two strains (**Figure 5**), demonstrating that there was no difference in the ability to circularize double-stranded DNA between the strains. These data suggest that the high AMT ability of the BW25113sm strain was specific for VirD2-driven transfer.

### Intact Structure of Plasmid DNA After VirD2-Driven Transfer to *E. coli*

**FIGURE 6:**
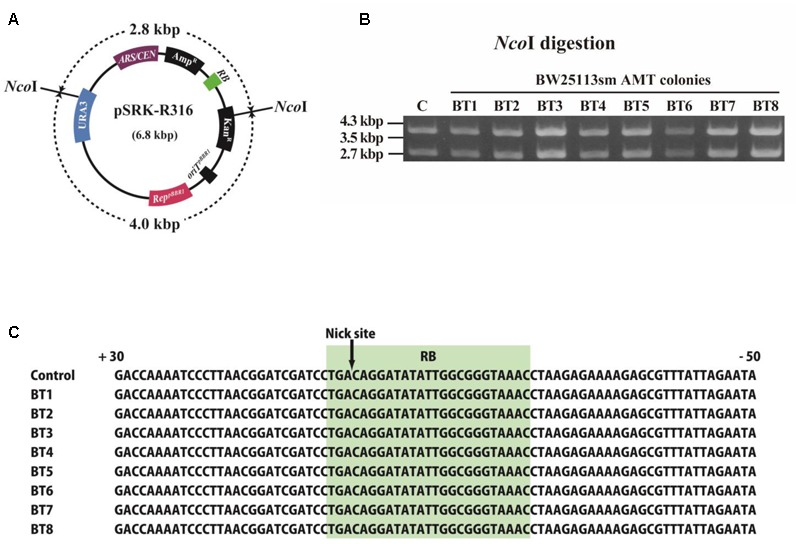
Structure of T-DNA circles extracted from *E. coli* transformant colonies. *E. coli* AMT offspring were obtained by co-cultivation with the donor strain EHA105 (pSRK-R316) and the recipient BW25113sm. **(A)** Physical map of pSRK-R316 showing the locations of two *Nco*I sites. **(B)** Extracted plasmid DNAs were digested by *Nco*I and separated by gel electrophoresis. Lane C: pSRK-R316, lanes 1 – 8: AMT colonies. **(C)** Alignment of *RB* junction sequences of plasmids extracted from the eight transformant colonies. The nucleotide sequence at the *RB* is shaded in light green.

The structure of pSRK-R316 after its AMT transfer to recipient *E. coli* cells was examined as demonstrated in **Figure 6**. VirD2-driven transfer was performed on BW25113sm, and then the plasmid DNAs were extracted from eight colonies. Restriction enzyme digestion of the plasmid DNAs suggested that the transferred plasmid DNAs retained their native structures (**Figures 6A,B**). Further analysis of the extracted plasmids confirmed that the nucleotide sequence at/around the *RB* was identical to that of pSRK-R316 (**Figure 6C**).

## Discussion

### Successful Model T-DNA Plasmid Transfer to Bacteria, and Its Impact

T-DNA was transmitted to a Gram-positive bacterium, *Streptomyces lividans*, from *Agrobacterium* via the VirB/D4 system (Kelly and Kado, 2002), and derivatives of the RSF1010 plasmid were mobilized to *Agrobacterium* (Beijersbergen et al., 1992). This paper has shown that two Gram-negative bacteria, *E. coli* and *A. tumefaciens*, are capable of receiving the model T-DNA plasmid pSRK-R316 from *Agrobacterium*. These data suggest that the VirB/D4 transfer apparatus has the fundamental potential to cover the domain Bacteria as the recipient range in AMT.

The model T-DNA plasmid pSRK-R316 contains an *RB* but no *LB*, just like conjugative and mobilizable plasmids including pAY205 possess an *oriT*. The transfer of pSRK-R316 depended strictly on VirD2 (**Figures 2–4** and **Table 3B**), and the plasmid DNA showed the same structure after transfer as the original plasmid DNA (**Figure 6**). These results suggest that the VirD2-driven transfer system retains the functionality of the ancestral conjugal transfer system.

The strain BW25113sm was the best recipient for VirD2-driven transfer of the model T-DNA plasmid among the *E. coli* strains we examined in this study. Various tools are available in the strain BW25113. Notably, systematic resources including mutants have been constructed using BW25113 and its near identical strain W3110 (Baba et al., 2006; Rajagopala et al., 2010), and their phenotypes in several conditions have been described. Such resources would assist the study of the molecular processes of DNA transfer in recipient cells.

### Characteristics of VirD2 Revealed in Reference to Mob

This study indicated that the fitness of Mob-driven AMT (transfer of pAY205) to recipient organisms is inverse to that of VirD2-driven AMT (transfer of pSRK-R316) (**Figure 2** and **Table 3**). High and very low frequencies of VirD2-driven AMT were observed in the transfer to yeast and bacterial strains, respectively. This result is reasonable if we consider that plants are the native target recipients for VirD2 and yeast belongs to the domain Eukaryota as do plants. Based on the high frequency of Mob-driven AMT of bacteria and the high frequency of VirD2-driven AMT of yeast via the same T4SS, we speculate that pSRK-R316 is transferred more abundantly to bacterial cells than was estimated based on the VirD2-driven AMT frequencies for bacterial recipient strains (**Table 3A**). The difference of AMT output productivity depending on host types is primarily attributable to the properties of the two relaxase proteins, and second to differences in the processes and interactions of the relaxases with recipient factors.

All data in this study show the superiority of Mob over VirD2 for plasmid transfer in bacteria. Though VirD2 has evolved from the relaxase for conjugation, we presume that VirD2 has adapted to function in plants so much that it has become weak at interacting with bacterial proteins.

### Insight Into the Roles of Relaxases, RecA and RAD Proteins in Plasmid Reception in Recipient Cells

It is noteworthy that in this study *recAΔ* mutation caused a 32-fold decrease in VirD2-driven AMT in *E. coli*, while the same mutation caused a threefold decrease in Mob-driven AMT. Though the latter value is tiny compared with the large decrease in VirD2-driven AMT, the value one-third of the wild type level might reflect a short transient exposure of the ssDNA to the recipient cytoplasm during Mob-driven transfer, and some role played by RecA.

RecA plays multiple roles in bacteria (Bell and Kowalczykowski, 2016). Primarily, RecA binds to single-stranded portions of damaged DNA and directs its repair, and upon binding to ssDNAs triggers the expression of a set of genes for DNA repair and recombination. RecA also plays a role in accepting exogenous ssDNA in competent *Bacillus subtilis*. The protein binds to a competency protein, GomGA, that is localized at the cell pole and imports exogenous DNAs (Kidane and Graumann, 2005; Kidane et al., 2012). RecA also associates with RecN, which attaches to the 3′-OH of ssDNA and might sequester the extreme end of the ssDNA within nucleoid structures (Sanchez and Alonso, 2005). The behavior of RecA in *B. subtilis* could represent a step in transformation for the inclusion of exogenous DNA into recipient genomic DNAs through recombination, and also suggest a role in conjugation.

Interestingly, the recipient yeast genes central to the HR process are also involved in AMT of yeast using a similar but different set of T-DNA plasmids. Rolloos et al. (2014) and Ohmine et al. (2016) performed yeast AMT using similar but different sets of model T-DNA plasmids having *RB* and *LB* borders. T-DNA circles were formed in the recipient yeast at high frequency. The yeast *RAD51* gene is a homolog of the bacterial *recA* gene (Shinohara et al., 1992; Krogh and Symington, 2004), and Rad52 helps Rad51 to perform strand exchange in yeast (Shinohara and Ogawa, 1998). The AMT efficiency is decreased by *rad51Δ* and *rad52Δ* (Rolloos et al., 2014; Ohmine et al., 2016). The defect caused by these mutations seems to not be in simple HR because the hyper HR mutation *srs2Δ* does not increase but decreases AMT as seriously as *rad52Δ* (Ohmine et al., 2016). We have data that show the yeast *srs2Δ* mutation also has a similar apparent negative effect on the transfer of pSRK-R316 (Kiyokawa, personal communication).

**FIGURE 7:**
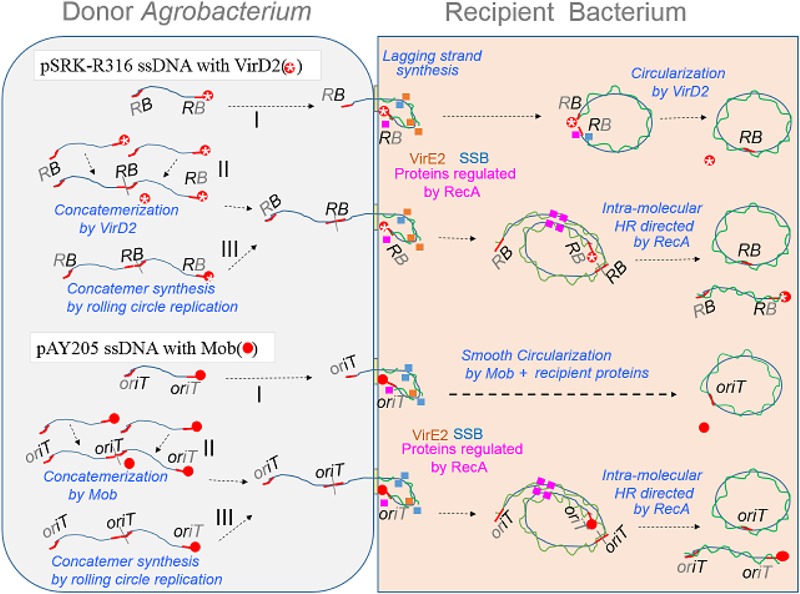
Schematic models of AMT of bacteria with an emphasis on the DNA structure of transfer intermediates, relaxase and ssDNA binding proteins (SSB and VirE2). ssDNA covalently linked with a relaxase protein, either Mob (filled red circle) or VirD2 (red circle marked with a star), is formed by the action of the relaxase on its target plasmid DNA. Rolling circle replication produces a monomer **(I)** molecule with a relaxase at each 5′-terminus. The nucleoprotein is mobilized via a VirB/D4 channel to a recipient bacterium. Upon entry of the nucleoprotein molecule, DNA polymerase starts lagging strand synthesis. Simultaneously, ssDNA portions are bound by ssDNA binding proteins, namely VirE2 from the donor, recipient SSB, RecA and proteins whose expression is enhanced by RecA. Mob catalyzes re-circularization at high efficiency, while VirD2 re-circularizes less efficiently in the recipient bacterial cell. The nucleoprotein molecule in the donor cell can also participate in another process that was previously proposed for yeast AMT (Rolloos et al., 2014). Two molecules are merged to form a concatemer linked with a relaxase **(II)**; this reaction releases a relaxase molecule. Rolling circle replication sometimes produces a concatemer **(III)** molecule having a relaxase at each 5’-terminus. The concatemers **(II,III)** enter the recipient bacterium and finally produce monomeric circles through HR directed by RecA.

We suppose that the ssDNAs from donor cells are bound by RecA and by some proteins whose expression requires RecA (Fernandez De Henestrosa et al., 2000), and that these proteins help VirD2 and Mob to re-circularize the ssDNAs (**Figure 7** pathway I). Because VirD2-driven yeast AMT and Mob-driven *E. coli* AMT were efficient, it is likely that the model T-DNA plasmid is easily transferred to *E. coli* cells. A plausible explanation for the low AMT efficiency in *E. coli* is that the plasmid circularization process that occurs through the DNA-joining activity of VirD2 (Pansegrau et al., 1993) proceeds only slowly in recipient *E. coli* cells, probably because of VirD2’s inability to associate with *E. coli* proteins, and therefore most transferred DNA molecules are degraded in the bacteria. In contrast, Mob can interact with recipient bacterial proteins better, and therefore perform AMT of bacteria efficiently, even though the *in vitro* ssDNA ligase ability of VirD2 (Pansegrau et al., 1993) looks much higher than that of Mob (Bhattacharjee and Meyer, 1991). Conversely, VirD2 is superior to Mob for yeast AMT, because VirD2 can interact well with several yeast proteins that are conserved among eukaryotes including plants.

In parallel with pathway I, which employs VirD2 and Mob proteins for the re-circularization in recipient cells, two other pathways are pictured in **Figure 7**. **Figure 7** pathway II involves concatemer formation through merging two monomers by VirD2 and Mob in donor *Agrobacterium* cells, and then circularization by HR in recipient cells, as proposed by Rolloos et al. (2014). The last model (**Figure 7** pathway III) involves no DNA-joining activity by any relaxase. In the formation of ssDNA, the cycle of monomeric ssDNA formation sometimes does not terminate and therefore generates multimeric forms of ssDNA, which could be turned into a monomer in recipient cells by HR.

## Author Contributions

YO conceived the study, performed most of the experiments, and wrote the draft manuscript. KK designed the study, performed most of the experiments, analyzed statistically, and finalized the manuscript. KY constructed plasmids, established and performed plasmid transfer experiments. SY provided strains and source plasmids, instructed the experiments, and finalized the manuscript. KM instructed the experiments, and finalized the manuscript. KS designed and instructed the whole body of the study. All authors read and approved the manuscript.

## Conflict of Interest Statement

The authors declare that the research was conducted in the absence of any commercial or financial relationships that could be construed as a potential conflict of interest.

## Footnotes

1 https://www.r-project.org/

## Abbreviations

AMT: *Agrobacterium*-mediated transformation
AS: acetosyringone
HR: homologous recombination
*LB*: left border of T-DNA
*RB*: right border of T-DNA
ss: single-strand
SSB: single-stranded DNA binding protein
T-DNA: transfer DNA region of Ti plasmids
T4SS: type IV secretion system
VirB/D4: channel composed of VirB proteins and VirD4 protein

## References

[B1] Alvarez-Martinez C. E. Christie P. J. (2009). Biological diversity of prokaryotic type IV secretion systems. *Microbiol. Mol. Biol. Rev.* 73 775–808. 10.1128/MMBR.00023-09 19946141 PMC2786583

[B2] Baba T. Ara T. Hasegawa M. Takai Y. Okumura Y. Baba M. (2006). Construction of *Escherichia coli* K-12 in-frame, single-gene knockout mutants: the Keio collection. *Mol. Syst. Biol.* 2:2006.0008. 10.1038/msb4100050 16738554 PMC1681482

[B3] Beijersbergen A. Dulk-Ras A. D. Schilperoort R. A. Hooykaas P. J. (1992). Conjugative transfer by the virulence system of *Agrobacterium tumefaciens*. *Science* 256 1324–1327. 10.1126/science.256.5061.1324 17736763

[B4] Bell J. C. Kowalczykowski S. C. (2016). RecA: regulation and mechanism of a molecular search engine. *Trends Biochem. Sci.* 41 491–507. 10.1016/j.tibs.2016.04.002 27156117 PMC4892382

[B5] Bevan M. (1984). Binary Agrobacterium vectors for plant transformation. *Nucleic Acids Res.* 12 8711–8721. 10.1093/nar/12.22.8711 6095209 PMC320409

[B6] Bhattacharjee M. K. Meyer R. J. (1991). A segment of a plasmid gene required for conjugal transfer encodes a site-specific, single-strand DNA endonuclease and ligase. *Nucleic Acids Res.* 19 1129–1137. 10.1093/nar/19.5.1129 1850512 PMC333791

[B7] Binns A. N. Beaupre C. E. Dale E. M. (1995). Inhibition of VirB-mediated transfer of diverse substrates from *Agrobacterium tumefaciens* by the IncQ plasmid RSF1010. *J. Bacteriol.* 177 4890–4899. 10.1128/jb.177.17.4890-4899.1995 7665465 PMC177262

[B8] Bohne J. Yim A. Binns A. N. (1998). The Ti plasmid increases the efficiency of *Agrobacterium tumefaciens* as a recipient in virB-mediated conjugal transfer of an IncQ plasmid. *Proc. Natl. Acad. Sci. U.S.A.* 95 7057–7062. 10.1073/pnas.95.12.7057 9618538 PMC22737

[B9] Boyer H. W. Roulland-Dussoix D. (1969). A complementation analysis of the restriction and modification of DNA in *Escherichia coli*. *J. Mol. Biol.* 41 459–472. 10.1016/0022-2836(69)90288-5 4896022

[B10] Brachmann C. B. Davies A. Cost G. J. Caputo E. Li J. Hieter P. (1998). Designer deletion strains derived from *Saccharomyces cerevisiae* S288C: a useful set of strains and plasmids for PCR-mediated gene disruption and other applications. *Yeast* 14 115–132. 10.1002/(SICI)1097-0061(19980130)14:2<115::AID-YEA204>3.0.CO;2-2 9483801

[B11] Bravo-Angel A. M. Gloeckler V. Hohn B. Tinland B. (1999). Bacterial conjugation protein MobA mediates integration of complex DNA structures into plant cells. *J. Bacteriol.* 181 5758–5765. 10482518 10.1128/jb.181.18.5758-5765.1999 PMC94097

[B12] Bundock P. den Dulk-Ras A. Beijersbergen A. Hooykaas P. J. (1995). Trans-kingdom T-DNA transfer from *Agrobacterium tumefaciens* to Saccharomyces cerevisiae. *EMBO J.* 14 3206–3214. 7621833 10.1002/j.1460-2075.1995.tb07323.x PMC394382

[B13] Christie P. J. Whitaker N. Gonzalez-Rivera C. (2014). Mechanism and structure of the bacterial type IV secretion systems. *Biochim. Biophys. Acta* 1843 1578–1591. 10.1016/j.bbamcr.2013.12.019 24389247 PMC4061277

[B14] De Vos G. Zambryski P. (1989). Expression of *Agrobacterium* nopaline-specific VirD1, VirD2, and VirC1 proteins and their requirement for T-strand production in *E. coli*. *Mol. Plant Microbe Interact.* 2 43–52. 10.1094/MPMI-2-043 2520160

[B15] Dube T. Kovalchuk I. Hohn B. Thomson J. A. (2004). *Agrobacterium tumefaciens*-mediated transformation of plants by the pTF-FC2 plasmid is efficient and strictly dependent on the MobA protein. *Plant Mol. Biol.* 55 531–539. 10.1007/s11103-004-1159-1 15604698

[B16] Fernandez De Henestrosa A. R. Ogi T. Aoyagi S. Chafin D. Hayes J. J. Ohmori H. (2000). Identification of additional genes belonging to the LexA regulon in *Escherichia coli*. *Mol. Microbiol.* 35 1560–1572. 10.1046/j.1365-2958.2000.01826.x 10760155

[B17] Firth N. Ippen-Ihler K. Skurray R. (1996). “Structure and function of the F factor and mechanism of conjugation,” in *Escherichia coli and Salmonella: Cellular and Molecular Biology* 2nd Edn ed. Neidhardt F. C. (Washington, DC: ASM Press) 2377–2401.

[B18] Frey J. Bagdasarian M. (1989). “The molecular biology of the IncQ plasmids,” in *Promiscuous Plasmids of Gram Negative Bacteria* ed. Thomas C. M. (London: Academic Press) 79–94.

[B19] Fullner K. J. (1998). Role of Agrobacterium virB genes in transfer of T complexes and RSF1010. *J. Bacteriol.* 180 430–434. 9440537 10.1128/jb.180.2.430-434.1998 PMC106903

[B20] Gelvin S. B. (2012). Traversing the cell: *Agrobacterium* T-DNA’s journey to the host genome. *Front. Plant Sci.* 3:52. 10.3389/fpls.2012.00052 22645590 PMC3355731

[B21] Grant S. G. Jessee J. Bloom F. R. Hanahan D. (1990). Differential plasmid rescue from transgenic mouse DNAs into *Escherichia coli* methylation-restriction mutants. *Proc. Natl. Acad. Sci. U.S.A.* 87 4645–4649. 10.1073/pnas.87.12.4645 2162051 PMC54173

[B22] Hood E. E. Murphy J. M. Pendleton R. C. (1993). Molecular characterization of maize extensin expression. *Plant Mol. Biol.* 23 685–695. 10.1007/BF00021524 8251623

[B23] Ippen-Ihler K. Skurray R. A. (1993). “Genetic organization of transfer-related determinants on the sex factor F and related plasmids,” in *Bacterial Conjugation* ed. Clewell D. B. (New York, NY: Springer) 23–52.

[B24] Kelly B. A. Kado C. I. (2002). *Agrobacterium*-mediated T-DNA transfer and integration into the chromosome of *Streptomyces lividans*. *Mol. Plant Pathol.* 3 125–134. 10.1046/j.1364-3703.2002.00104.x 20569318

[B25] Khan S. R. Gaines J. Roop R. M. Farrand S. K. (2008). Broad-host-range expression vectors with tightly regulated promoters and their use to examine the influence of TraR and TraM expression on Ti plasmid quorum sensing. *Appl. Environ. Microbiol.* 74 5053–5062. 10.1128/Aem.01098-08 18606801 PMC2519271

[B26] Kidane D. Ayora S. Sweasy J. B. Graumann P. L. Alonso J. C. (2012). The cell pole: the site of cross talk between the DNA uptake and genetic recombination machinery. *Crit. Rev. Biochem. Mol. Biol.* 47 531–555. 10.3109/10409238.2012.729562 23046409 PMC3490228

[B27] Kidane D. Graumann P. L. (2005). Intracellular protein and DNA dynamics in competent *Bacillus subtilis* cells. *Cell* 122 73–84. 10.1016/j.cell.2005.04.036 16009134

[B28] Kiyokawa K. Yamamoto S. Sakuma K. Tanaka K. Moriguchi K. Suzuki K. (2009). Construction of disarmed Ti plasmids transferable between *Escherichia coli* and Agrobacterium species. *Appl. Environ. Microbiol.* 75 1845–1851. 10.1128/Aem.01856-08 19181833 PMC2663228

[B29] Kiyokawa K. Yamamoto S. Sato Y. Momota N. Tanaka K. Moriguchi K. (2012). Yeast transformation mediated by Agrobacterium strains harboring an Ri plasmid: comparative study between GALLS of an Ri plasmid and virE of a Ti plasmid. *Genes Cells* 17 597–610. 10.1111/j.1365-2443.2012.01612.x 22686249

[B30] Krogh B. O. Symington L. S. (2004). Recombination proteins in yeast. *Annu. Rev. Genet.* 38 233–271. 10.1146/annurev.genet.38.072902.091500 15568977

[B31] Lacroix B. Tzfira T. Vainstein A. Citovsky V. (2006). A case of promiscuity: *Agrobacterium’s* endless hunt for new partners. *Trends Genet.* 22 29–37. 10.1016/j.tig.2005.10.004 16289425

[B32] Lanka W. Pansegrau W. (1999). “Genetic exchange between microorganisms,” in *Biology of the Prokaryotes* eds Lengeler J. W. Drews G. Schlegel H. G. (Stuutgart: Georg Thieme Verlag) 386–415.

[B33] Lawley T. Frost L. Wilkins B. M. (2004). “Bacterial conjugation in Gram-negative bacteria,” in *Plasmid Biology* eds Funnell B. Phillips G. (Washington, DC: ASM Press) 203–226.

[B34] Lawley T. D. Klimke W. A. Gubbins M. J. Frost L. S. (2003). F factor conjugation is a true type IV secretion system. *FEMS Microbiol. Lett.* 224 1–15. 10.1016/S0378-1097(03)00430-0 12855161

[B35] Lessl M. Lanka E. (1994). Common mechanisms in bacterial conjugation and Ti-mediated T-DNA transfer to plant cells. *Cell* 77 321–324. 10.1016/0092-8674(94)90146-5 8181052

[B36] Motohashi K. (2015). A simple and efficient seamless DNA cloning method using SLiCE from *Escherichia coli* laboratory strains and its application to SLiP site-directed mutagenesis. *BMC Biotechnol.* 15:47. 10.1186/s12896-015-0162-8 26037246 PMC4453199

[B37] Nishikawa M. Suzuki K. Yoshida K. (1992). DNA integration into recipient yeast chromosomes by trans-kingdom conjugation between *Escherichia coli* and *Saccharomyces cerevisiae*. *Curr. Genet.* 21 101–108. 10.1007/BF00318467 1568253

[B38] Ohmine Y. Satoh Y. Kiyokawa K. Yamamoto S. Moriguchi K. Suzuki K. (2016). DNA repair genes *RAD52* and *SRS2*, a cell wall synthesis regulator gene *SMI1*, and the membrane sterol synthesis scaffold gene *ERG28* are important in efficient *Agrobacterium*-mediated yeast transformation with chromosomal T-DNA. *BMC Microbiol.* 16:58. 10.1186/s12866-016-0672-0 27038795 PMC4818910

[B39] Okegawa Y. Motohashi K. (2015). Evaluation of seamless ligation cloning extract preparation methods from an *Escherichia coli* laboratory strain. *Anal. Biochem.* 486 51–53. 10.1016/j.ab.2015.06.031 26133399

[B40] Pansegrau W. Lanka E. (1991). Common sequence motifs in DNA relaxases and nick regions from a variety of DNA transfer systems. *Nucleic Acids Res.* 19:3455. 10.1093/nar/19.12.3455 1648208 PMC328351

[B41] Pansegrau W. Schoumacher F. Hohn B. Lanka E. (1993). Site-specific cleavage and joining of single-stranded DNA by VirD2 protein of *Agrobacterium tumefaciens* Ti plasmids: analogy to bacterial conjugation. *Proc. Natl. Acad. Sci. U.S.A.* 90 11538–11542. 10.1073/pnas.90.24.11538 8265585 PMC48019

[B42] Rajagopala S. V. Yamamoto N. Zweifel A. E. Nakamichi T. Huang H. K. Mendez-Rios J. D. (2010). The *Escherichia coli* K-12 ORFeome: a resource for comparative molecular microbiology. *BMC Genomics* 11:470. 10.1186/1471-2164-11-470 20701780 PMC3091666

[B43] Rolloos M. Dohmen M. H. Hooykaas P. J. van der Zaal B. J. (2014). Involvement of Rad52 in T-DNA circle formation during *Agrobacterium tumefaciens*-mediated transformation of Saccharomyces cerevisiae. *Mol. Microbiol.* 91 1240–1251. 10.1111/mmi.12531 24460832

[B44] Sanchez H. Alonso J. C. (2005). *Bacillus subtilis* RecN binds and protects 3′-single-stranded DNA extensions in the presence of ATP. *Nucleic Acids Res.* 33 2343–2350. 10.1093/nar/gki533 15849320 PMC1084328

[B45] Sawahel W. Sastry G. Knight C. Cove D. (1993). Development of an electro-transformation system for *Escherichia coli* DH10B. *Biotechnol. Tech.* 7 261–266. 10.1007/Bf00150895

[B46] Schäfer A. Tauch A. Jäger W. Kalinowski J. Thierbach G. Pühler A. (1994). Small mobilizable multi-purpose cloning vectors derived from the *Escherichia coli* plasmids pK18 and pK19: selection of defined deletions in the chromosome of *Corynebacterium glutamicum*. *Gene* 145 69–73. 10.1016/0378-1119(94)90324-7 8045426

[B47] Scheiffele P. Pansegrau W. Lanka E. (1995). Initiation of *Agrobacterium tumefaciens* T-DNA processing. Purified proteins VirD1 and VirD2 catalyze site- and strand-specific cleavage of superhelical T-border DNA in vitro. *J. Biol. Chem.* 270 1269–1276. 10.1074/jbc.270.3.1269 7836390

[B48] Scherzinger E. Kruft V. Otto S. (1993). Purification of the large mobilization protein of plasmid RSF1010 and characterization of its site-specific DNA-cleaving/DNA-joining activity. *Eur. J. Biochem.* 217 929–938. 10.1111/j.1432-1033.1993.tb18323.x 8223650

[B49] Scholz P. Haring V. Wittmann-Liebold B. Ashman K. Bagdasarian M. Scherzinger E. (1989). Complete nucleotide sequence and gene organization of the broad-host-range plasmid RSF1010. *Gene* 75 271–288. 10.1016/0378-1119(89)90273-4 2653965

[B50] Shinohara A. Ogawa H. Ogawa T. (1992). Rad51 protein involved in repair and recombination in *S. cerevisiae* is a RecA-like protein. *Cell* 69 457–470. 10.1016/0092-8674(92)90447-K 1581961

[B51] Shinohara A. Ogawa T. (1998). Stimulation by Rad52 of yeast Rad51-mediated recombination. *Nature* 391 404–407. 10.1038/34943 9450759

[B52] Sikorski R. S. Hieter P. (1989). A system of shuttle vectors and yeast host strains designed for efficient manipulation of DNA in *Saccharomyces cerevisiae*. *Genetics* 122 19–27. 2659436 10.1093/genetics/122.1.19 PMC1203683

[B53] Simon R. Priefer U. Pühler A. (1983). A broad host range mobilization system for *in vivo* genetic engineering: transposon mutagenesis in gram negative bacteria. *Nat. Biotechnol.* 1 784–791. 10.1038/nbt1183-784

[B54] Simonsen L. Gordon D. M. Stewart F. M. Levin B. R. (1990). Estimating the rate of plasmid transfer: an end-point method. *J. Gen. Microbiol.* 136 2319–2325. 10.1099/00221287-136-11-2319 2079626

[B55] Smillie C. Garcillan-Barcia M. P. Francia M. V. Rocha E. P. de la Cruz F. (2010). Mobility of plasmids. *Microbiol. Mol. Biol. Rev.* 74 434–452. 10.1128/MMBR.00020-10 20805406 PMC2937521

[B56] Stahl L. E. Jacobs A. Binns A. N. (1998). The conjugal intermediate of plasmid RSF1010 inhibits *Agrobacterium tumefaciens* virulence and VirB-dependent export of VirE2. *J. Bacteriol.* 180 3933–3939. 9683491 10.1128/jb.180.15.3933-3939.1998 PMC107378

[B57] Suzuki K. Tanaka K. Yamamoto S. Kiyokawa K. Moriguchi K. Yoshida K. (2009). “Ti and Ri plasmids,” in *Microbial Megaplasmids* ed. Schwartz E. (Heidelberg: Springer Verlag) 133–147.

[B58] Vogel A. M. Das A. (1992). Mutational analysis of *Agrobacterium*-tumefaciens virD2: tyrosine 29 is essential for endonuclease activity. *J. Bacteriol.* 174 303–308. 10.1128/jb.174.1.303-308.1992 1309520 PMC205709

[B59] Ward E. R. Barnes W. M. (1988). VirD2 protein of *Agrobacterium tumefaciens* very tightly linked to the 5′ end of T-strand DNA. *Science* 242 927–930. 10.1126/science.242.4880.927

[B60] Wong J. J. Lu J. Glover J. N. (2012). Relaxosome function and conjugation regulation in F-like plasmids - a structural biology perspective. *Mol. Microbiol.* 85 602–617. 10.1111/j.1365-2958.2012.08131.x 22788760

[B61] Yamamoto S. Uraji M. Tanaka K. Moriguchi K. Suzuki K. (2007). Identification of pTi-SAKURA DNA region conferring enhancement of plasmid incompatibility and stability. *Genes Genet. Syst.* 82 197–206. 10.1266/ggs.82.197 17660690

[B62] Zambryski P. C. (1992). Chronicles from the Agrobacterium-plant cell DNA transfer story. *Annu. Rev. Plant Physiol. Plant Mol. Biol.* 43 465–490. 10.1146/annurev.pp.43.060192.002341

[B63] Zupan J. R. Citovsky V. Zambryski P. (1996). Agrobacterium VirE2 protein mediates nuclear uptake of single-stranded DNA in plant cells. *Proc. Natl. Acad. Sci. U.S.A.* 93 2392–2397. 10.1073/pnas.93.6.2392 8637884 PMC39807

